# Machine-learning guided Venom Induced Dermonecrosis Analysis tooL: VIDAL

**DOI:** 10.1038/s41598-023-49011-6

**Published:** 2023-12-08

**Authors:** William Laprade, Keirah E. Bartlett, Charlotte R. Christensen, Taline D. Kazandjian, Rohit N. Patel, Edouard Crittenden, Charlotte A. Dawson, Marjan Mansourvar, Darian S. Wolff, Thomas Fryer, Andreas H. Laustsen, Nicholas R. Casewell, José María Gutiérrez, Steven R. Hall, Timothy P. Jenkins

**Affiliations:** 1 Department of Applied Mathematics and Computer Science, Technical University of Denmark, Kongens Lyngby, Denmark; 2 Centre for Snakebite Research and Interventions, Liverpool School of Tropical Medicine, Liverpool, UK; 3 Department of Biotechnology and Biomedicine, Technical University of Denmark, Kongens Lyngby, Denmark; 4 Instituto Clodomiro Picado, Facultad de Microbiología, Universidad de Costa Rica, San José, Costa Rica; 5 Lancaster Medical School and Biomedical & Life Sciences, Lancaster University, Lancaster, UK

**Keywords:** Skin manifestations, Image processing, Machine learning

## Abstract

Snakebite envenoming is a global public health issue that causes significant morbidity and mortality, particularly in low-income regions of the world. The clinical manifestations of envenomings vary depending on the snake's venom, with paralysis, haemorrhage, and necrosis being the most common and medically relevant effects. To assess the efficacy of antivenoms against dermonecrosis, a preclinical testing approach involves in vivo mouse models that mimic local tissue effects of cytotoxic snakebites in humans. However, current methods for assessing necrosis severity are time-consuming and susceptible to human error. To address this, we present the Venom Induced Dermonecrosis Analysis tooL (VIDAL), a machine-learning-guided image-based solution that can automatically identify dermonecrotic lesions in mice, adjust for lighting biases, scale the image, extract lesion area and discolouration, and calculate the severity of dermonecrosis. We also introduce a new unit, the dermonecrotic unit (DnU), to better capture the complexity of dermonecrosis severity. Our tool is comparable to the performance of state-of-the-art histopathological analysis, making it an accessible, accurate, and reproducible method for assessing dermonecrosis in mice. Given the urgent need to address the neglected tropical disease that is snakebite, high-throughput technologies such as VIDAL are crucial in developing and validating new and existing therapeutics for this debilitating disease.

## Introduction

Snakebite envenoming is a major public health problem, especially in low-income regions of the world^1^. Indeed, it is responsible for substantial morbidity and mortality, particularly in the impoverished areas of the tropics and subtropics, such as sub-Saharan Africa, South and Southeast Asia, Papua New Guinea, and Latin America^2–4^. Whilst accurate estimates are difficult to obtain, it is believed that between 1.8 and 2.7 million people worldwide are envenomed each year, resulting in 80,000–140,000 deaths and around 400,000 victims left with permanent sequelae^5–7^. Notably, snake venoms are highly complex and comprise a wide range of toxins that differ across families, genera, and species. Consequently, the clinical manifestations and pathophysiological effects of envenomings can vary greatly depending on which snake species was responsible for the snakebite. Here, paralysis, haemorrhage, and necrosis constitute some of the most common and/or medically relevant effects^8^. This also results in the need to test different venoms for the preclinical validation of antivenom efficacy. To complicate matters further, the severity of a given envenoming is significantly affected by the amount of venom injected and the anatomical location of the bite^9^. When a new antivenom is developed, or an existing one is introduced to a new geographical setting, it needs to undergo preclinical efficacy testing; this involves the assessment of its neutralising capacity against the lethal effects of venom(s) in mice^10,11^, but it may also involve a diverse set of tests to assess neutralisation of other relevant toxic effects, such as necrosis within the skin (i.e., dermonecrosis). This pathology is predominately caused by cytotoxic 3FTxs (three-finger toxins), SVMPs (snake venom metalloproteinases), and PLA_2_s (phospholipase A_2_s), which induce significant tissue damage by disrupting cell membranes, breaking down extracellular matrix, and inducing inflammation^1^. Venom induced dermonecrosis often results in surgical intervention, e.g. debridement or amputation of the affected limb^12–14^, and frequently results in loss of limb function or permanent disability.

The underlying mechanism by which snake venoms induce dermonecrosis, the characterisation of necrotising toxins, and their varying severities have, for a long time, presented a key area of fundamental and translational research within the field of Toxinology. As necrosis typically affects cutaneous and muscle tissues in snakebite victims, these are the two types of tissue most studied using in vivo mouse models to mimic the local tissue effects of cytotoxic snakebites in human victims^15^. Necrosis within the muscle tissue (myonecrosis) is primarily assessed by injecting venom or toxins into the gastrocnemius muscle of mice and assessing the necrosis-inducing potential via the quantification of the extent of muscle damage by histological analysis (i.e. haemotoxylin & eosin (H&E)-staining) or by quantifying the plasma activity of creatine kinase (CK)^16^. Alternatively, dermonecrosis caused by snake venoms is tested using methods initially described by Theakston et al.^17^ in which mice are injected intradermally with sub-lethal doses of venom (with or without venom-inhibiting treatments) to induce tissue damage within the skin’s layers, and after 72 h, the mice are euthanised and the width and height of the venom-induced lesions are measured using callipers. While able to assess the impact of a treatment on cytotoxic effects of venoms, this method has limitations including susceptibility to human error and does not consider lesion severity (i.e., a light lesion [characterised by hyperplasia of epidermis] and dark lesion [characterised by a disruption and loss of the epidermis] of the same size but with different intensities would be regarded as equally severe using this method). In an attempt to better differentiate lesion severity, one option is to analyse and quantify dermonecrosis severity within each skin layer using histopathological analysis of H&E-stained lesion cross-sections^18^. However, this method is time consuming, requires analysis and quantification by trained pathologists, and is also susceptible to human error.

Given the current drive towards addressing the neglected tropical disease that is snakebite, major efforts are being undertaken into defining pathologies, understanding which toxins are responsible, as well as testing and developing new and existing therapeutics. Thus, high throughput, accurate, and reproducible technologies are key in ensuring that these efforts are as time- and cost-efficient as possible. Therefore, in this article, we present a new and accessible machine-learning guided solution, i.e., the Venom Induced Dermonecrosis Analysis tooL, VIDAL (GitHub: https://github.com/laprade117/VIDAL/tree/VIDAL; DOI: 10.5281/zenodo.10229152). Here, we trained a machine learning algorithm to automatically identify dermonecrotic lesions in mice using photography images, adjust for lighting biases, scale the image, extract lesion area and discolouration, and calculate the severity of dermonecrosis. We also propose a new unit to better capture the complexity of dermonecrosis severity, i.e., the dermonecrotic unit (DnU). We validate the utility of this tool for quantitatively defining dermonecrosis using samples derived from animal models of envenoming and demonstrate our tool is comparable to the performance of the current state of the art histopathological analysis.

## Results

**Figure 1:**
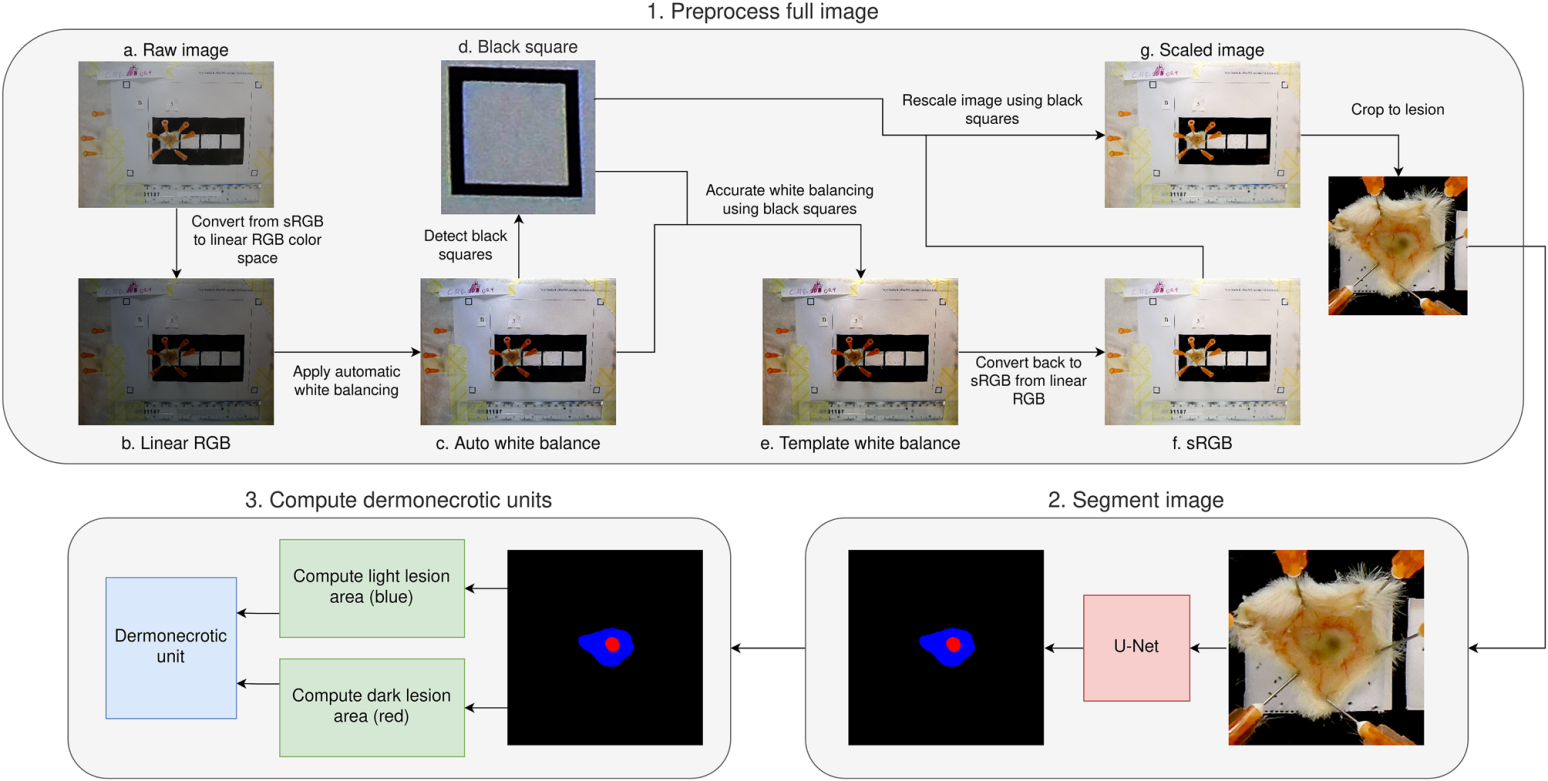
Overview of the workflow for VIDAL. First, the raw image is imported and converted from standard red, green, blue (sRGB) to linear RGB. Thereafter, the image is white balanced and subsequently further white balanced using the colour of the paper detected via the scaling squares. In parallel, the image is rescaled using the same squares. This processed image is then used for segmentation and automatic identification of the necrotic lesions. Together, this information is used to compute the dermonecrotic lesion area and differentiate between a severe dark dermonecrotic lesion (red) and a less severe light region of local tissue damage (blue). The areas of each dermonecrotic and light region of local tissue damage are then combined based on our histopathologically determined (c.f. “Segmentation is able to identify and distinguish between light and dark lesions”) weighting of 2.019 to 1 into dermonecrotic units (DnU).

In this study, we present our VIDAL image analysis tool trained on 193 sample images sourced from diverse experiments, including murine dermonecrotic lesions induced by the intradermal injection of venoms from *Crotalus atrox*, *Echis ocellatus*, *Bothrops asper*, *Naja nigricollis, Naja pallida*, and *Bitis arietans* which provide a diversity of lesion sizes, colours, and intensities (Fig. 1). All venoms used induced dermonecrosis in mice, as observed macroscopically, and the intensity of dermonecrosis was dependent upon the snake species from which the venom was collected and the venom dose injected. To illustrate the appropriateness of our methods, we present histopathological analyses for nine example lesions that are subsequently also assessed with VIDAL, i.e., three healthy tissue controls only injected with PBS (C1_A, C1_B, C2_A); three light lesions caused by West African *N. nigricollis* (57 µg; L1_A, L1_B,) and *N. pallida* (25 µg; L2_A) venom; and three dark lesions caused by East African *N. nigricollis* (63 µg; D1_A, D1_B) and West African *N. nigricollis* (57 µg; D2_A) venom. These venoms were known to cause distinct light and dark lesions in the skin.

### Histopathological analysis of H&E-stained sections of venom-induced lesions demonstrate clear differences in light and dark lesions

**Figure 2:**
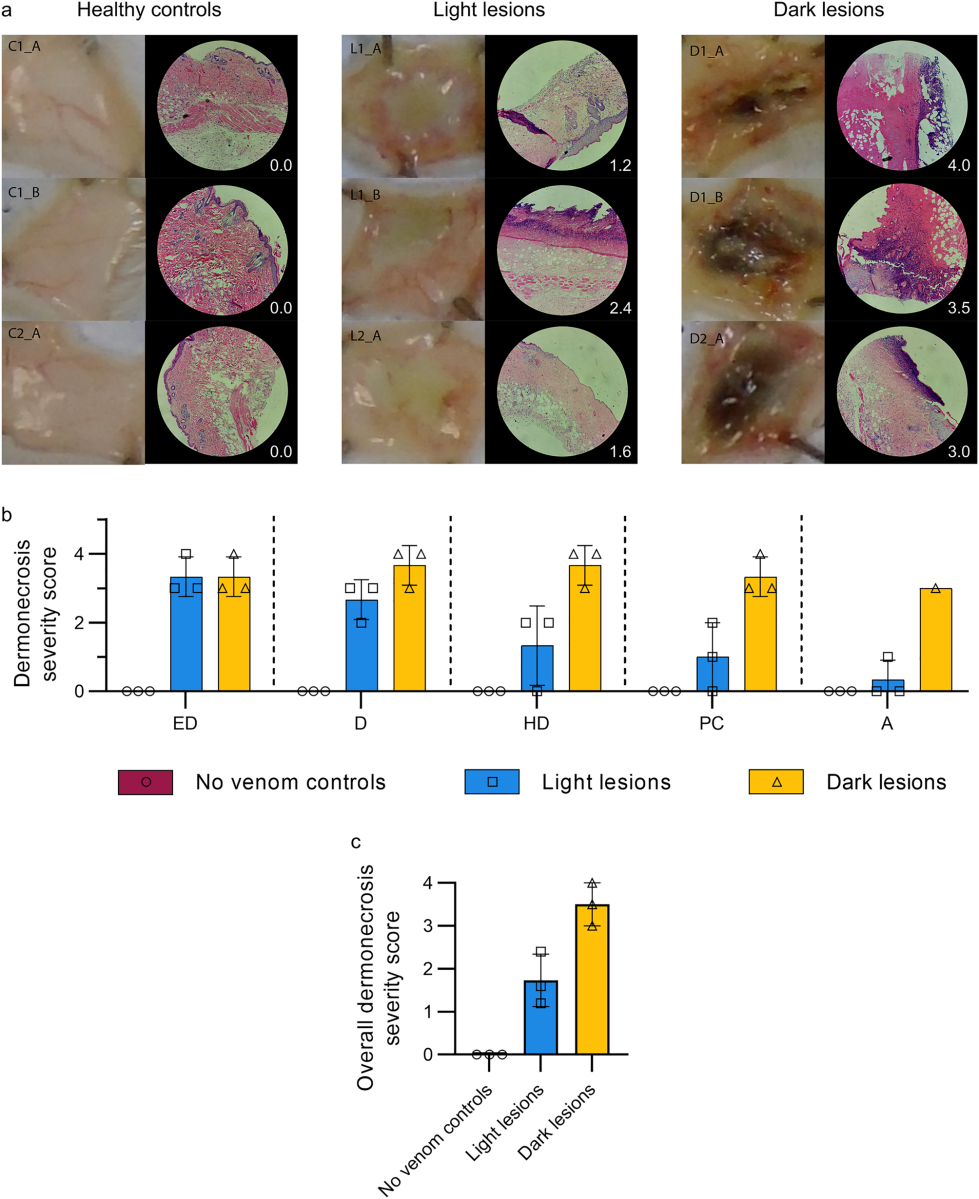
Histopathological analysis of mouse skin cross-sections of healthy tissue, light lesions, and dark lesions confirms dark lesions are more severely dermonecrotic than light lesions. Four µm thick H&E sections from formalin-fixed, paraffin-embedded tissue were previously prepared from the dermal injection sites of mice 72 h after being ID-injected with PBS (C1_A, C1_B, C2_A) or dermonecrotic snake venoms, *i.e.,* three light lesions caused by West African *N. nigricollis* (57 µg; L1_A, L1_B,) and *N. pallida* (25 µg; L2_A); three dark lesions caused by East African *N. nigricollis* (63 µg; D1_A, D1_B) and West African *N. nigricollis* (57 µg; D2_A). An author with expertise in pathological analysis (JMG) scored, between 0 and 4, the percentage of necrosis that was visible within each skin layer, where 0 = 0%, 2 = 25–50%, 3 = 50–75%, and 4 = 75–100%, based on previously developed methodology^19^. The highest recorded score per skin layer was taken as the measure of maximum severity it reached, and the mean score of all layers per sample taken as its overall dermonecrosis severity score. (**a**) Macroscopic and representative 100×-magnified H&E-stained images with their associated overall dermonecrosis severity scores in the bottom right-hand corners, can be seen for (left) healthy tissue controls (C1_A, C1_B, C2_A), (middle) light lesions (L1_A, L1_B, L2_A), and (right) dark lesions (D1_A, D1_B, D2_A). (**b**) Bar graphs summarising the measured level of dermonecrosis in each skin layer [epidermis (ED), dermis (D), hypodermis (HD), panniculus carnosus (PC), and adventitia (A)], and (**c**) mean overall dermonecrosis severity score for each lesion or control tissue sample. All H&E-stained tissue images used in the measurements can be seen in Fig. S4.

Skin tissue samples from mice injected with PBS showed the characteristic histological features of normal skin, including epidermis, dermis, hypodermis, panniculus carnosus, and adventitia layers. In contrast, the skin layers of mice injected with snake venoms showed various degrees of damage, depending on the type and dose of venom. In the case of tissue sections collected from macroscopically dark lesions, the extent of damage was more pronounced than in macroscopically light lesions. Using the dermonecrosis scoring system developed previously for quantifying lesion severity in H&E-stained lesion cross-sections^19^, the healthy tissue, light lesions, and dark lesions each received mean overall dermonecrosis severity scores of 0.00, 1.73, and 3.50, respectively (Figs. 2, S1). The dark versus light lesion severity weighting of 2.02 was then calculated by dividing the dark by the light lesion mean overall dermonecrosis severity score.

### White balancing is able to normalise for different lighting conditions

**Figure 3:**
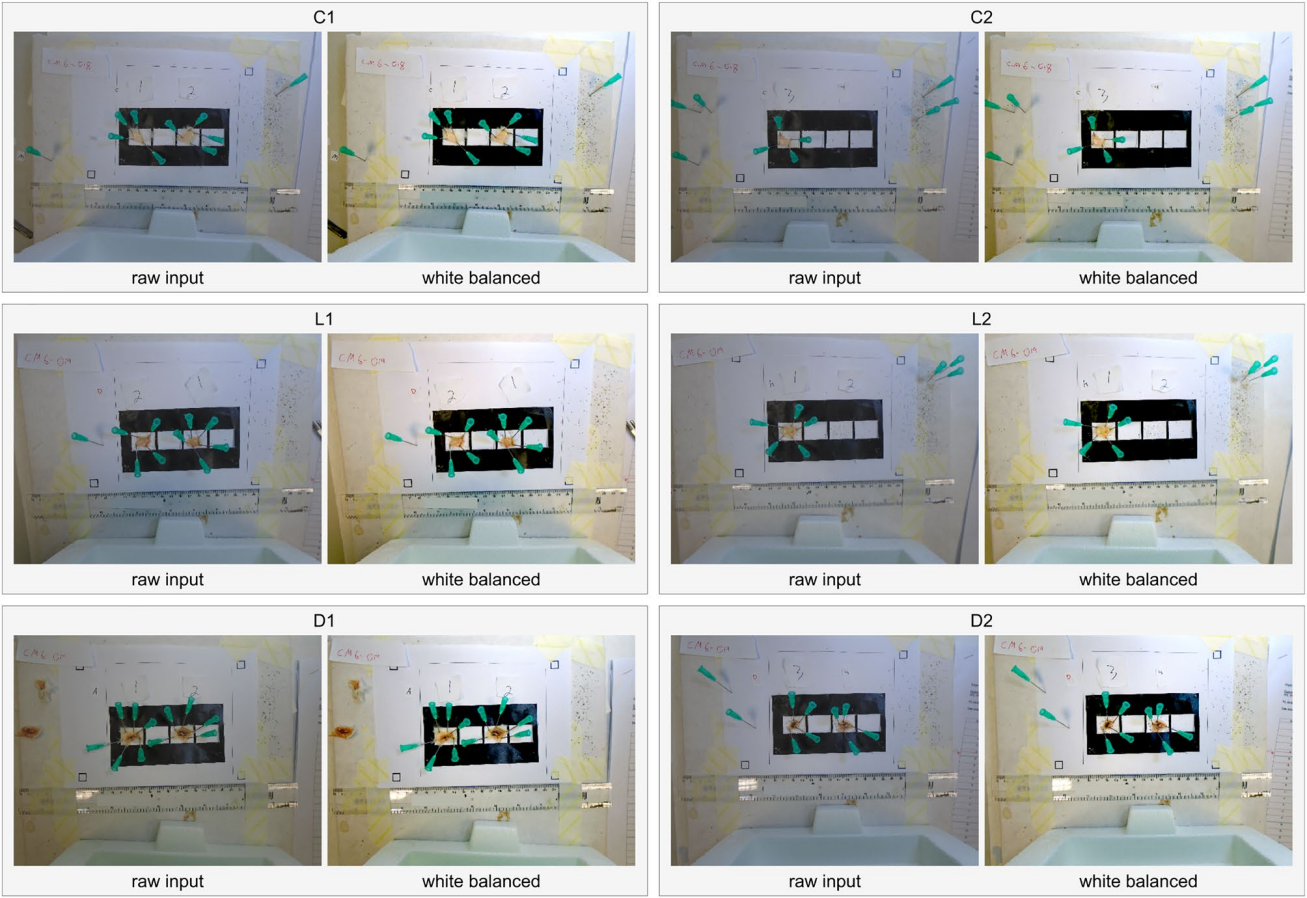
An overview of VIDAL’s automatic white balancing output across the 9 example lesions. Included were three healthy tissue controls only injected with PBS (C1_A, C1_B, C2_A); three light lesions caused by West African *N. nigricollis* (57 µg; L1_A, L1_B,) and *N. pallida* (25 µg; L2_A); three dark lesions caused by East African *N. nigricollis* (63 µg; D1_A, D1_B) and West African *N. nigricollis* (57 µg; D2_A).

The tool automatically applies white balancing to account for potential lighting variations, ensuring consistent results across images. The white balancing function operates effectively, yielding comparable outcomes (Fig. 3).

**Figure 4:**
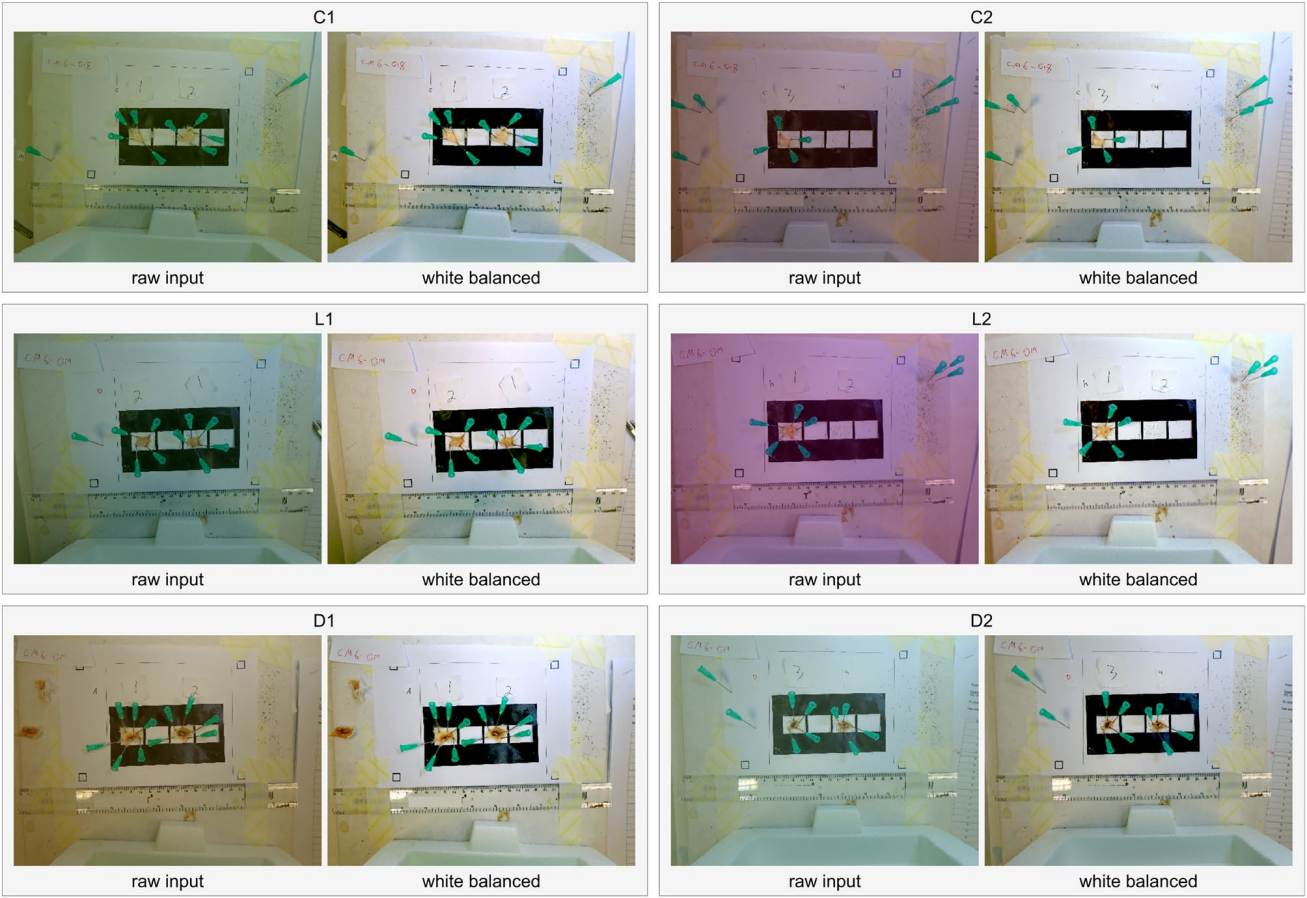
Stress testing VIDAL’s automatic white balancing capacity. The colours of the six images were randomly manipulated to assess white balancing performance. Included were three healthy tissue controls only injected with PBS (C1_A, C1_B, C2_A); three light lesions caused by West African *N. nigricollis* (57 µg; L1_A, L1_B,) and *N. pallida* (25 µg; L2_A); three dark lesions caused by East African *N. nigricollis* (63 µg; D1_A, D1_B) and West African *N. nigricollis* (57 µg; D2_A). _A always corresponds to the first lesion (left to right), _B to the second and _C to the third.

In order to conduct a more rigorous evaluation of the white balancing feature's ability to adapt to challenging lighting conditions, we introduced image manipulations by randomly simulating various colours. In each simulation, the tool successfully restored an image that was similar in quality (Fig. 4).

### Scaling is able to ensure scale normalisation of images

To determine scale, the tool first uses a standard template matching algorithm to locate the black squares in the corners of the paper template in each image. Using the known scale of these black squares, we can then compute the scale of the images (in pixels per mm) and resize the images to a target scale. We use a target of 5 pixels per mm as this allows us to fit the entire lesion nicely into a 256 × 256 patch that we can feed into the U-Net segmentation model directly (Table 1).

**Table 1:** Table outlining the input dimensions, detected scale, target scale, and output dimensions across the 9 example lesions. Included were three healthy tissue controls only injected with PBS (C1_A, C1_B, C2_A); three light lesions caused by West African *N. nigricollis* (57 µg; L1_A, L1_B,) and *N. pallida* (25 µg; L2_A); three dark lesions caused by East African *N. nigricollis* (63 µg; D1_A, D1_B) and West African *N. nigricollis* (57 µg; D2_A).

	C1	C2	L1	L2	D1	D2
Input dimensions (pixel × pixel)	3864 × 5152	3864 × 5152	3864 × 5152	3864 × 5152	3864 × 5152	3864 × 5152
Detected scale (pixels per mm)	9.9247	10.0936	12.8996	11.0422	13.8578	10.5688
Target scale (pixels per mm)	5	5	5	5	5	5
Output dimensions (pixel × pixel)	1947 × 2596	1914 × 2552	1498 × 1997	1750 × 2333	1394 × 1859	1828 × 2437

### Segmentation is able to identify and distinguish between light and dark lesions

**Figure 5:**
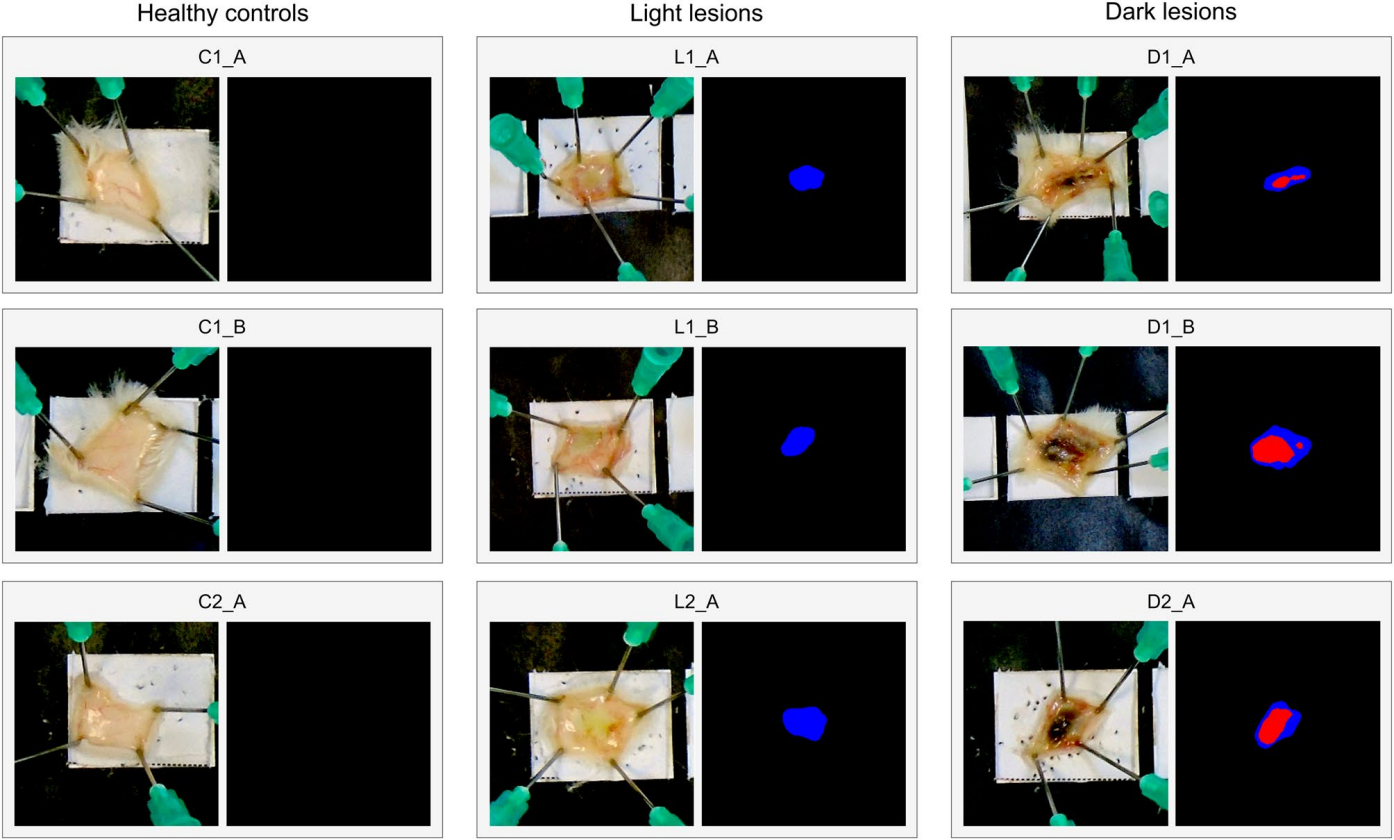
Segmentation of all necrotic lesions across the 9 lesions assessed in the histopathological analysis. The blue regions denote light necrosis and the red regions signify dark necrosis. Included were three healthy tissue controls only injected with PBS (C1_A, C1_B, C2_A); three light lesions caused by West African *N. nigricollis* (57 µg; L1_A, L1_B,) and *N. pallida* (25 µg; L2_A); three dark lesions caused by East African *N. nigricollis* (63 µg; D1_A, D1_B) and West African *N. nigricollis* (57 µg; D2_A).

To automatically identify lesion areas, the tool uses a U-Net segmentation model to segment the lesions located at user-defined positions in the image. Overall, an average MCC score of 0.7644 and an average F1 (Dice) score of 0.8738 were achieved, and we were able to predict 99.98% of the pixels correctly across 25 runs (Fig. 5).

### Dermonecrotic units present an easy and representative readout for lesion severity

To assess the severity of each lesion, the tool automatically computes the real area and DnU for each mouse skin excision in all of the images (Table 2).

**Table 2:** Dermonecrotic units and lesion sizes for the six example test images.

Results									
Lesion	C1_A	C1_B	C2_A	L1_A	L1_B	L2_A	D1_A	D1_B	D2_A
Light area (mm^2^)	0.00	0.00	0.00	41.96	43.28	71.00	33.08	63.04	41.24
Dark area (mm^2^)	0.00	0.00	0.00	0.00	0.00	0.00	12.84	55.80	44.60
DnU	0.00	0.00	0.00	41.96	43.28	71.00	58.98	175.59	131.20

Included were three healthy tissue controls only injected with PBS (C1_A, C1_B, C2_A); three light lesions caused by West African *N. nigricollis* (57 µg; L1_A, L1_B,) and *N. pallida* (25 µg; L2_A); three dark lesions caused by East African *N. nigricollis* (63 µg; D1_A, D1_B) and West African *N. nigricollis* (57 µg; D2_A).

### The tool’s graphical user interface is simple and accessible

**Figure 6:**
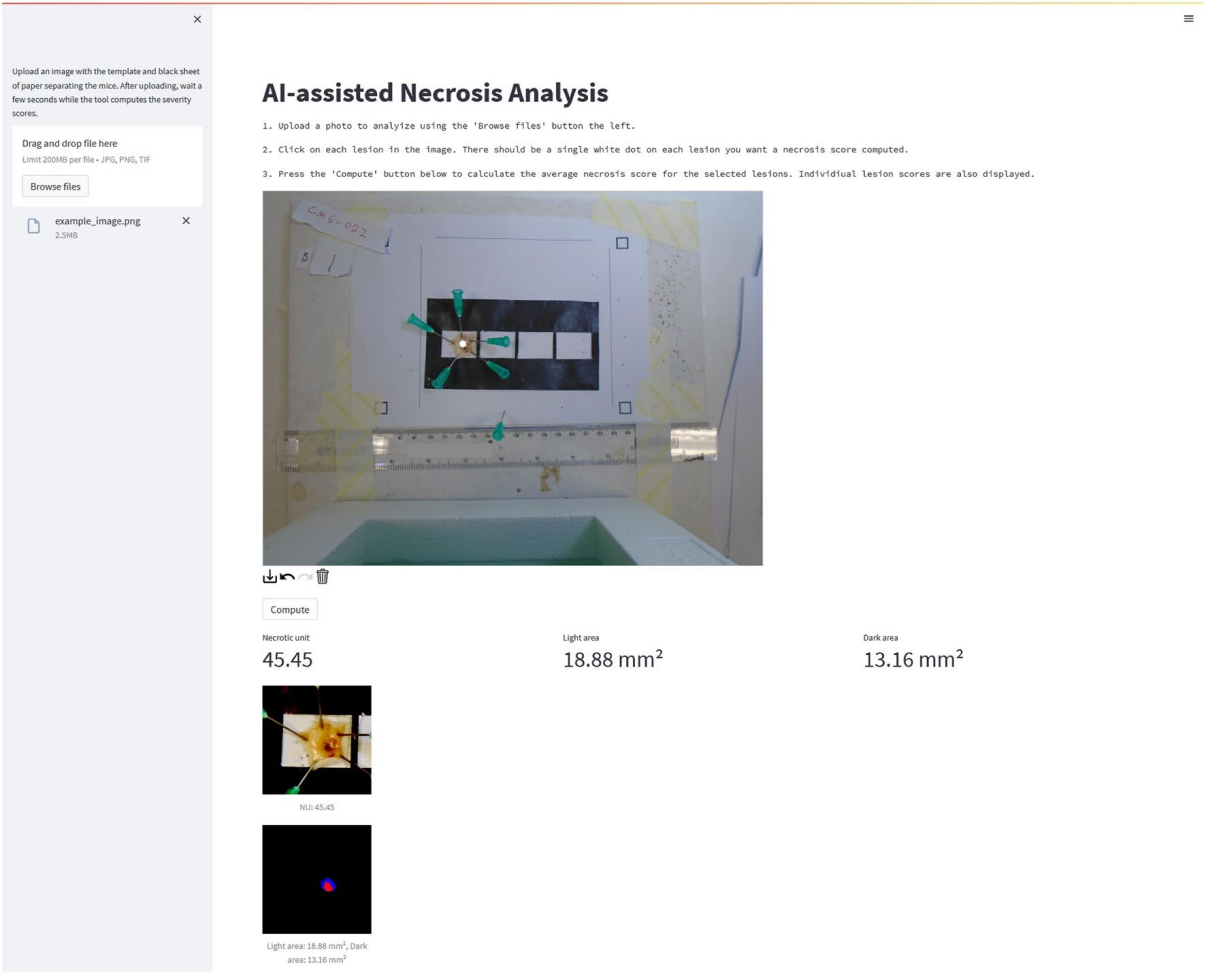
Image of VIDAL’s web interface. By uploading an image file of the experiment, the tool will, within seconds, provide the white balanced reference image, the individual lesions it detected, and how it decided to segment them, as well as all relevant data on lesion areas and DnUs.

To ensure accessibility and easy implementation of VIDAL across research, production, and quality control laboratories, a graphical user interface was developed (DOI: 10.5281/zenodo.10229152). Our tool can be used to quickly upload an image and receive statistics on the lesion area, luminance, and DnU for each mouse in the image (Fig. 6).

## Discussion

Snake venom-induced dermonecrosis presents one of the most severe clinical manifestations of envenoming by viperid and some elapid species and is responsible for a substantial proportion of the estimated 400,000 permanent sequelae induced by snakebite envenomings globally every year^1^. Furthermore, current antivenoms are largely ineffective in preventing the rapid destruction of tissues that snake venoms may cause, unless administered very soon after the bite^1^. Therefore, a thorough understanding of snake venom dermonecrosis and the neutralising potential of current treatments, as well as next generation therapeutics for this pathophysiological manifestation, is crucial^12–14,20^. However, to date, no rapid and robust dermonecrosis assays exist that can be used to help create such a thorough understanding. Indeed, current assessments involve the manual measurements of dermonecrotic lesions with callipers^19–21^ or the use of manual lesion outlining on transparent plastic sheets and calculation of the encompassed area with millimetric paper^22^. Both of these methods have inherent limitations, as they can only assess entirely spherical areas precisely (which dermonecrotic lesions seldomly are) and are susceptible to potential unconscious bias and human error. Additionally, both existing methods entirely fail to evaluate the severity of the dermonecrotic damage, *i.e.,* a light lesion and dark lesion of the same size would be considered equally severe. This shortcoming can be overcome via the use of histopathological analysis, which is accurate, but low-throughput and requires training as well as specialised equipment^18,19^.

Until recently, the assessment of snake venom haemorrhage was faced with the same issues. Therefore, we first developed a simple computational tool to improve accuracy of quantifying haemorrhage severity, which has since been implemented by different groups within the field^23–31^. This encouraged us to build a more sophisticated machine learning guided tool that further increased the accuracy, accessibility, and reproducibility of snake venom haemorrhage assessment^32^.

In this study, we applied a similar approach to the area of dermonecrosis and developed and evaluated an easy-to-use, rapid, and accurate method for the evaluation of snake venom-induced dermonecrosis in a rodent skin model. This model can both measure the exact size of the lesions and provide a severity score based on the presence of light (less severe lesions) versus dark (more severe lesions) lesions. Thereby, a more accurate and rapid measure of dermonecrosis compared to current techniques is achieved. Images generated from envenoming by a variety of viper and elapid venoms that were known to induce dermonecrosis were used to ensure that the results were representative. Histopathological analyses confirmed the different extents of overall skin damage in light versus dark macroscopic lesions, as judged by the more severe pathological alterations observed in various layers of the skin in the latter. We further built a fully automated analysis pipeline, supported by vision AI (U-Net). Our tool, VIDAL, efficiently and accurately evaluated a diverse set of training images that encompassed varying levels of dermonecrotic lesion severity. Throughout our experimentation, we consistently observed reliable white balancing, precise scaling, and accurate segmentation. To further examine the white balancing feature, we artificially manipulated the images to simulate different lighting conditions and colour settings, mimicking scenarios where different cameras were used by different users. Our tool effectively adjusted the white balance of these manipulated images, demonstrating its robustness in experimental settings. To minimize animal usage during the initial validation phase, we performed segmentation on a limited dataset. Nevertheless, U-Net has consistently displayed its robustness in previous studies^33–36^, even when trained on small datasets, which was the case in our study. The segmentation results consistently aligned with expert opinions, even when the images were artificially manipulated to simulate various laboratory and lighting conditions, thus creating a more challenging environment for testing image quality.

In order to ensure maximum accessibility and seamless integration into existing workflows, we have developed a user-friendly web tool with a graphical user interface (GUI). This tool enables users to perform quick and precise analyses of dermonecrotic lesions. For example, by utilising a smartphone to capture and upload an image of a murine dermonecrotic lesion, an accurate measure of its severity can be determined within minutes. This remarkable reduction in analysis time from hours to just a few minutes has significantly enhanced efficiency. Additionally, the user-friendly nature of the tool greatly increases accessibility, enabling us to provide a standardized solution that can be utilised in laboratories without extensive training or prior knowledge of lesion assessment requirements. Thereby, this tool may help strengthen the area of toxicovenomics, *i.e.*, the study of venom proteomes in relation to their functional toxicity^37^, by improving standardisation and harmonisation of toxicity data across venoms, models, and labs. In turn, this may help provide a clearer overview of the key toxin targets that need to be neutralised by antivenoms, and guide the development of novel envenoming therapeutics^38–40^.

Whilst the tool brings many advantages, it does come with some of the same limitations as our prior tool^32^. These include potential decreases in white balancing accuracy in very poorly or unevenly lit environments, as well as a heavily damaged template sheet. Additionally, lesions that include significant haemorrhaging are sometimes poorly recognised, potentially complicating the analysis of necrosis induced by some viper venoms; we therefore recommend that users aim to clean such lesions and assess these images on a case-by-case basis to ensure that the program accurately detects their lesions. Finally, though only an issue for certain venoms, the tool could present false positives for some of the very light lesions (i.e., secondary skin damage) due to similarity to the mouse skin colour; this is primarily an issue in lower resolution images. Though VIDAL should be applicable to a broad number of necrosis inducing venoms as it has been trained on lesions induced by 6 different snake species (both vipers and elapids), it has only been validated on cobra envenomings to date.

## Conclusion

With the Venom Induced Dermonecrosis Analysis tooL VIDAL, we introduce a rapid and robust new method for the automated assessment of venom-induced dermonecrosis in mice by implementing sophisticated machine learning based image analysis approaches. This method eliminates the risk of human biases in assessing lesion areas and increases the speed of analysis substantially. We hope that this will be of utility for the study of dermonecrotic toxins and venoms and provide researchers with an extra tool to be implemented in the assessment of neutralising efficacy of antivenoms and inhibitors.

## Methods

Images of murine dermonecrotic lesions and preparation of Haematoxylin & Eosin (H&E)-stained slides used to develop the described tool were derived from previously completed experiments from separate parallel studies associated with the development of new snakebite therapeutics. Only the blinded images from the skins of mice injected with *Crotalus atrox*, *Echis ocellatus*, *Bothrops asper*, *Naja nigricollis, Naja pallida*, and *Bitis arietans* venoms, representing diverse lesion sizes, colours and intensities, were selected as training set data for the VIDAL algorithm. All images used in its creation have been made available in DOI: 10.5281/zenodo.10229159. Though not part of this study, methodological details of the type of in vivo experiments performed and H&E-slide preparation are summarised below, since the images used for training VIDAL were derived from these experiments.

### In vivo dermonecrosis model of envenoming

All animal experiments were conducted using protocols approved by the Animal Welfare and Ethical Review Boards of the Liverpool School of Tropical Medicine and the University of Liverpool and were performed in pathogen-free conditions under licensed approval (PPL #P58464F90) of the UK Home Office and in accordance with the Animal [Scientific Procedures] Act 1986 and institutional guidance on animal care as well as the ARRIVE guidelines. Animals (18–20 g [4–5 weeks old], male, CD-1 mice, Charles River, UK) were acclimatised for at least one week before experimentation with their health monitored daily. Mice were grouped in cages of five, with room conditions of approximately 22 °C at 40–50% humidity, with 12/12 h light cycles, and given ad lib access to CRM irradiated food (Special Diet Services, UK) and reverse osmosis water in an automatic water system. Mice were housed in specific-pathogen free facilities in Techniplast GM500 cages containing Lignocell bedding (JRS, Germany), Sizzlenest zigzag fibres as nesting material (RAJA), and supplied with environmental enrichment materials. Experimental design was based upon WHO-recommended envenoming protocols and the dermonecrosis methods were based on the Minimum Necrotizing Dose (MND) principles originally described in Theakston and Reid^17,29^. Venom treatments per mouse included (country of origin, dose): *Crotalus atrox* (USA, 100 µg), *Echis ocellatus* (Nigeria, 39 µg), *Bothrops asper* (Cost Rica, 150 µg), *Naja nigricollis* (Tanzania, 63 µg; or Nigeria, 57 µg)*, Naja pallida* (Tanzania, 25 µg), or *Bitis arietans* (Nigeria, 64 µg); control mice were injected with venom-free vehicle control. All treatment doses were diluted using PBS to a volume of 50 µL and incubated at 37 °C for 30 min, then kept on ice for no more than 3 h until the mice were ID injected. For dose delivery, mice were briefly anesthetised using inhalational isoflurane (4% for induction of anaesthesia, 1.5–2% for maintenance) and ID-injected in the shaved rear quadrant on the dorsal side of the flank skin with the 50 µL treatments. Experimenters at the time of injections were not blinded to the venoms used in each mouse so that they knew what snake species-specific signs of systemic envenoming to watch for as this would meet our previously defined humane endpoint; however, all annotators involved in the training of VIDAL were blinded, with images randomised prior to annotating. The animals were observed three times daily until 72 h post-injection to check for symptoms of systemic envenoming or excessive external lesion development (> 10 mm in diameter), which would have necessitated early termination of the animal due to reaching a humane endpoint defined by the animal ethics licence. At the end of the experiments the mice were euthanised using rising concentrations of CO_2_, after which the skin surrounding the injection site was dissected and internal skin lesions measured with callipers and photographed on the standardised printout sheet (described below). The outcome measures were the development, size, and severity of dermonecrotic lesions. Cross-sections of the skin lesions were further dissected and preserved in formalin for mounting on microscopy slides for downstream histopathological analysis. Each individual mouse was considered a separate experimental unit (“n”), with five mice allocated per treatment group as per previous intradermal (ID) venom injection studies^29^ and allocated randomly into each treatment group. In total 193 images, each from a separate mouse, were used in the creation of VIDAL, none of which were excluded.

### Preparation and histopathological analysis of H&E-stained sections of venom-induced lesions

Skin samples underwent tissue processing using a Tissue-Tek VIP (vacuum infiltration processor) overnight before being embedded in paraffin (Ultraplast premium embedding medium, Solmedia, WAX060). Next, 4 µm paraffin sections were cut on a Leica RM2125 RT microtome, floated on a water bath, and placed on colour slides (Solmedia, MSS54511YW) or poly-lysine slides (Solmedia MSS61012S) to dry. For haematoxylin & eosin (H&E) staining, slides were dewaxed in xylene and rehydrated through descending grades of ethanol (100%, 96%, 85%, 70%) to distilled water before being stained in haematoxylin for 5 min, “blued” in tap water for 5 min, then stained in eosin for 2 min. Slides were then dehydrated through 96% and 100% ethanol to xylene and cover slipped using DPX (Cellpath SEA-1304-00A). Haematoxylin (Atom Scientific, RRBD61-X) and Eosin (TCS, HS250) solutions were made up in house.

### Scoring of H&E-stained sections of venom-induced lesions

Brightfield images of the H&E-stained skin cross-section slides were taken with an Echo Revolve microscope (Settings: 10× magnification; LED: 100%; Brightness: 30; Contrast: 50; Colour balance: 50), with at least three images taken per skin cross-section. Dermonecrosis within each skin layer of each of the nine tissue samples was scored using methods outlined by Hall et al*.*^19^. In brief, the severity of dermonecrosis within each skin layer (epidermis, dermis, hypodermis, panniculus carnosus, and adventitia) was scored between 0 and 4 by a blinded experimenter. A score of 0 represented 0% of the layer within the image being affected, 1 represented up to 25%, 2 represented between 25 and 50%, 3 represented between 50 and 75%, and 4 being the most severe and representing > 75% of the skin layer. An overall dermonecrosis score was then calculated from the mean of the resulting scores obtained for the various layers. A more severe pattern of tissue damage was observed histologically in the dark-lesions as compared to the light lesions. Therefore, to take this difference into account, the mean dermonecrosis score of the dark-lesions (severe necrosis) was divided by that of the light lesions (less severe tissue damage) to calculate a ‘dark lesion severity adjustment score’, which was determined to be 2.02 (see details in the “Results” section).

### Printout sheet

To allow for standardised analysis of the dermonecrotic lesions, as well as to support the image analysis algorithms, we used the same A4 printout sheet as in our prior publication^32^ (c.f. Supplementary Fig. 1|A4 printout template to be used for dermonecrosis assays), which the tissue samples were placed on (Figs. S2, S3).

### Description of machine learning guided approach of quantifying dermonecrotic activity

We next trained a machine learning algorithm to automatically identify dermonecrotic lesions, adjust for lighting biases, scale the image, extract the dermonecrotic lesion area and discolouration, and calculate the DnUs. This was then implemented in a tool coined VIDAL (Fig. 1), for which we also prepared standard operating procedures (Fig. S4).

### White balancing, scaling, and segmentation

First, the input images were white balanced and scaled, as described in our prior publication^32^. Thereafter, to identify and segment the dermonecrotic lesions, we applied a deep learning method based on the U-Net architecture^33^, which we have previously also applied to snake venom induced haemorrhage identification^32^. Changes from the original U-Net architecture include replacing the deconvolution layers in the expanding path with bilinear upsampling, followed by a 2 × 2 convolution, adding batch normalisation layers and using padding in each convolutional layer to preserve image dimensions^41,42^.

Our dataset consisted of 193 training images taken (each with up to three lesions) with a Sony DSC W-800 camera. Each image contains 1–3 lesions displaying varying amounts of necrotic damage with both the light and dark necrotic regions in each image annotated. To limit annotator bias, each image was annotated by 4 different annotators resulting in 4 masks per image, each mask containing three classes (background/no lesion, light lesion, and dark lesion). For evaluating performance, we set aside 20% of the images at random as our held-out test set and performed fivefold cross-validation on the remaining 80% of the images, evaluating model performance on the held-out test set. This process was repeated 5 times to avoid test-set bias.

At the time of training, the images were split into samples of size 256 × 256 pixels and fed into the model in batches of 32 samples. Batches were created such that each sample has a 50% chance of having a masked section of dermonecrotic tissue according to at least one annotator. The mask used for training was sampled from the set of annotators at random. Data augmentations include flips, rotations, noise, blurring, sharpening, distortions, brightness, contrast, hue, and saturation adjustments. They were selected to simulate the possible variation in both the lighting environment as well as account for different built-in post-processing implementations in different types of cameras.

The models were trained using the Adam optimizer and with a learning rate of 0.0001 for 180 epochs. We used a loss function based upon a combination of the Mathews correlation coefficient (MCC) and cross-entropy^43^.

### Calculation of dermonecrotic units and minimum dermonecrotic dose

Snake venom induced dermonecrosis primarily manifests itself in two distinct macroscopic appearances: 1. a severe dark dermonecrotic lesion, and 2. a less severe light region of local tissue damage. To compute the dermonecrotic severity of a given lesion, we quantify the area of both pathologies (1 = light lesion and 2 = dark lesion) and then combined them via a weighted sum using our in vitro determined (c.f. “Segmentation is able to identify and distinguish between light and dark lesions”) weighting of 2.019 to 1 into DnUs.

$$DnU = type\_2 + 2.019*type\_1$$

### Implementation in GUI

Using Streamlit (https://github.com/streamlit/streamlit) and localtunnel (https://github.com/localtunnel/localtunnel) with Google Colab, a simple web-based application to automatically analyse images was developed DOI: 10.5281/zenodo.10229152 (https://github.com/laprade117/VIDAL). A web-based application seems to be an efficient way to quickly analyse data while working in the lab^32^. Users can take a photo with a smartphone and upload it to the web-based tool (accessible via a smartphone browser) for an immediate result (Fig. S4).

### Supplementary Information

Supplementary Information.

## Data Availability

The tool and sample images have been made available DOI: 10.5281/zenodo.10229152 and DOI: 10.5281/zenodo.10229159 , as well as via GitHub (https://github.com/laprade117/VIDAL; https://github.com/laprade117/VIDAL-Experiments).
